# Age-related changes in plasma levels of BDNF in Down syndrome patients

**DOI:** 10.1186/1742-4933-7-2

**Published:** 2010-01-25

**Authors:** Giada Dogliotti, Emanuela Galliera, Federico Licastro, Massimiliano M Corsi

**Affiliations:** 1 Department of Human Morphology and Biomedical Sciences "Città Studi", Laboratory of Clinical Pathology, University of Milan, Milan, Italy; 2 Department of Experimental Pathology, Laboratory of Immunology, University of Bologna, Bologna, Italy; 3 Laboratory of Biotechnological Applications, IRCCS Istituto Galeazzi, Milan, Italy

## Abstract

**Background:**

The prevalence of coronary artery diseases is low among Down Syndrome (DS) patients and they rarely die of atherosclerotic complications. Histopathological investigations showed no increase in atherosclerosis, or even a total lack of atherosclerotic changes, in DS

**Aim:**

The aim of our study is to investigate the relationship between age and brain-derived neurotrophic factor (BDNF) levels in Down Syndrome (DS).

**Subjects and methods:**

Three groups of DS patients were studied: the first consisted of 23 children (age 2-14 years); the second of 14 adults (age 20-50 years), the third group of 13 elderly persons (>60 years) and a controls group of 20 healthy patients (age 15-60 years).

The analytes of interest were quantified using a biochip array analyzer (Evidence^®^, Randox Ltd., Crumlin, UK).

**Results:**

Plasma BDNF was higher in DS patients than in controls and there was a significant age-related increase. Serum levels of IL-6 and MCP-1 were also higher in DS children and adults, but not in older patients, than in healthy control. High levels of circulating BDNF may protect DS patients from the clinical complications of atherosclerosis. However, the striking drop in peripheral BDNF levels with age might predispose these patients to clinical manifestations of dementia in later life.

## Introduction

The prevalence of coronary artery diseases is low among Down Syndrome (DS) patients and they rarely die of atherosclerotic complications [1]. Histopathological investigations showed no increase in atherosclerosis, or even a total lack of atherosclerotic changes, in DS [2]. Therefore, in spite of some classical biochemical risk factors for atherosclerosis, its clinical manifestation is low in DS. The reasons remain unclear, but recent studies have reported the potential importance of neurotrophins, such as nerve growth factor (NGF) and brain-derived neurotrophic factor (BDNF), in atherosclerosis and related disorders [3]. In particular, in DS patients interplay between NGF and inflammatory molecules IL-6 and MCP-1, have been described [4]. Brain-derived neurotrophic factor (BDNF) belongs to the neurotrophins family of proteins which, besides their neurotrophic functions, enhance survival and activity of a large number of non-neuronal cells [5,6]. BDNF is involved in mental retardation phenotype of DS. The phenotype of Down syndrome, trisomy of chromosome 21, is hypothesized to be produced by the increased expression due to gene dosage of normal chromosome 21 genes, which affects the regulation and function of several proteins (ELK, CREB, ER, GR) and BDNF, which are involved in certain facets of learning, memory and behavior that are abnormal in DS or mouse models [7]. This loss of regulation may be particularly significant in the etiology of neurodegenerative diseases that have a significant impact on the aging brain such as AD, Parkinson's disease, and autoimmune diseases [8,9]. It is of interest that DS patients have a high risk of developing Alzheimer's disease (AD) [4]. Although the neuropathological characteristics appear similar, it is not known whether the early-onset dementia seen in individuals with DS originates from the same biological mechanisms as in AD. Aging is a relevant factor affecting BDNF's ability to protect neuronal activity, but age related effects on BDNF function in non neuronal cells, in particular in atherosclerosis, still remain unclear.

The aim of this study was to evaluate a possible role of circulating BDNF in DS and its relationship with IL-6 and MCP-1 in DS patients of different ages. BDNF might be a protective biomarker for the clinical manifestation of atherosclerosis in DS.

## Subjects and methods

### Subjects

Three groups of DS patients were studied: the first consisted of 23 children (age 2-14 years); the second of 14 adults (age 20-50 years), the third group of 13 elderly persons (>60 years) and a control group of 30 healthy patients (age 2-65 years). All DS patients were assessed by clinical examination and karyotype analysis; they had mild and variable degrees of mental retardation, no other pathological conditions at the time of the study, and were in good health. The project was approved by the University of Milan Ethics Committee and by the Fondazione Antoniana of Bologna, Italy.

### Methods

Blood samples were collected from DS patients. Plasma was obtained by centrifugation, transferred into coded plastic tubes, rapidly frozen and stored at -20°C until analysis. The analytes of interest were quantified using a biochip array analyzer (Evidence^®^, Randox Ltd., Crumlin, UK). A biochip is a solid substrate where each specific ligand (antibodies) is spotted on discrete test regions. After an immuno-enzymatic reaction, each spot generates a chemiluminescent signal on the array which is captured by a charge-coupled camera (CCD-camera) and converted by image processing software to provide results comparable with calibration curves.

## Results

**Figure 1:**
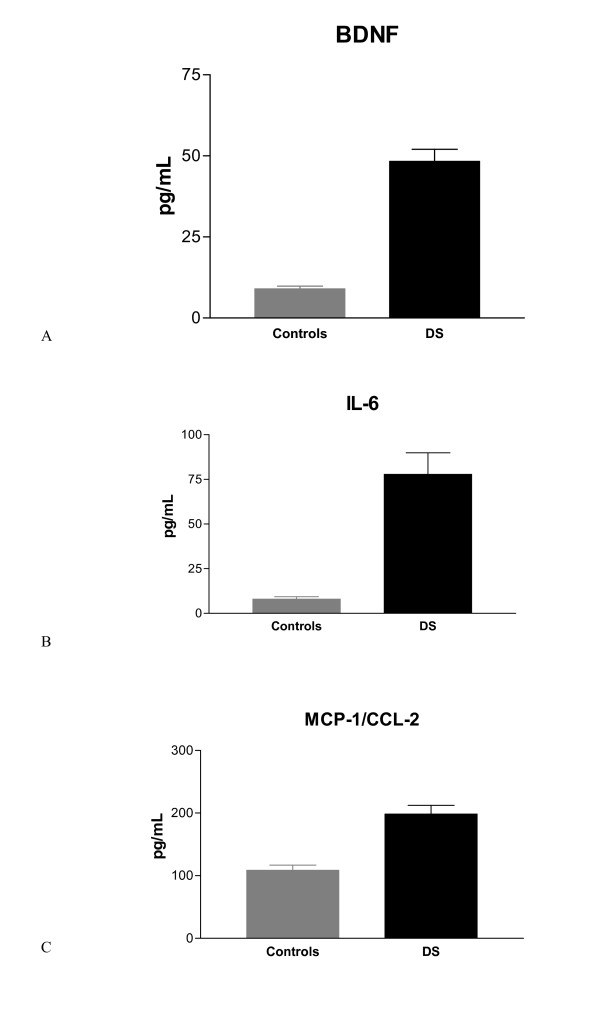
**A Graphical representation of BDNF levels in DS patients compared healthy subject**. B Graphical representation of IL-6 levels in DS patients compared healthy subject. C Graphical representation of MCP-1/CCL-2 levels in DS patients compared healthy subject.

Our data show a statistically increase of serum BDNF levels (fig. 1A) in Down's syndrome patients (48.28 ± 24.14 SD pg/mL), with an age range between 6 and 50 years, compared to healthy controls (8.96 ± 2.13 SD pg/mL, p = 0.0003) with similar age range. On the same patients two molecules involved in inflammatory processes, IL-6 and MCP-1, were studied. Fig. 1B shows a significant increase of IL-6 levels in Down's syndrome patients (77.75 ± 65.07 SD pg/mL) compared to controls (7.94 ± 3.38 SD pg/mL with a p = 0.0140). Similarly serum level of MCP-1, shown in fig 1C, increases statistically in Down's syndrome patients (198.44 ± 94.20 SD pg/mL) compared to healthy controls (108.55 ± 25.25 SD pg/mL with a p = 0.0067).

**Figure 2:**
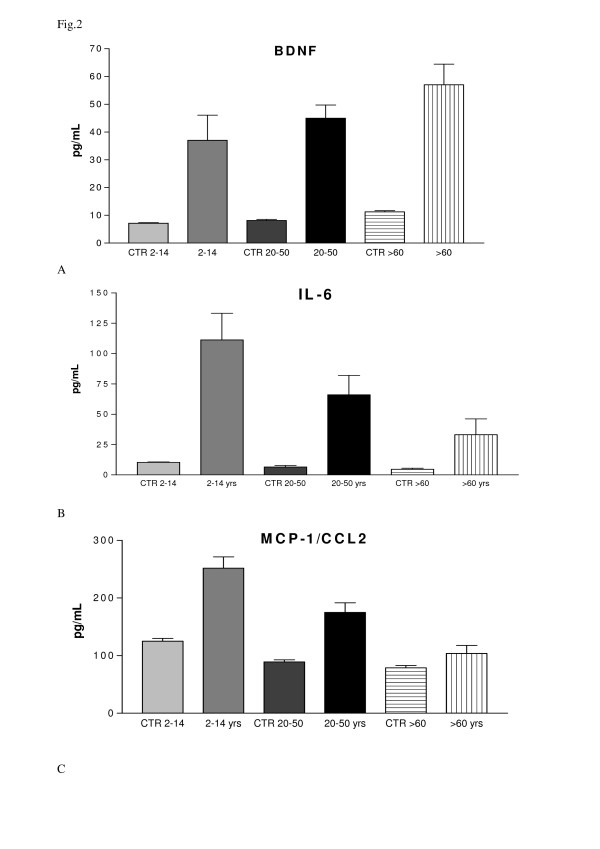
**A Graphical representation of BDNF levels in controls and DS patients subdivided in different age-range**. B Graphical representation of IL-6 levels in controls and DS patients subdivided in different age-range C Graphical representation of MCP-1/CCL-2 levels in controls and DS patients subdivided in different age-range.

Fig 2A shows that BDNF level is statistically significatively higher than control subjects of the same age range (children Down's syndrome patients 36.94 ± 22.15 SD pg/mL, children control 7.178 ± 0.468, p < 0.05; adult Down's syndrome patients 44.98 ± 21.34 SD pg/mL, adult control 8.14 ± 1.274 SD pg/mL, p < 0.001; old Down's syndrome patients 69.83 ± 22.15 SD pg/mL, old control 11.245 ± 1.035 SD pg/mL, p < 0.001). Among Down's syndrome patients, BDNF level increases in old (>60 years old) patients (69.83 ± 22.15 SD pg/mL) compared to both children (2-14 years old, 36.94 ± 22.15 SD pg/mL with p < 0.01) and adults (20-50 years old) Down's syndrome patients (44.98 ± 21.34 SD pg/mL with p < 0.01). Opposite trend is observed for IL-6 serum levels. Data shown in fig 2B indicate a statistically decrease in old Down's syndrome patients (33.17 ± 31.77 SD pg/mL) compared to children Down's syndrome patients (111.28 ± 76.02 SD pg/mL with p < 0.05) and a not statistically decrease compared to adults Down's syndrome patients (66.05 ± 50.38 SD pg/mL). Also in this case DS patients levels differ form control subject of the same age (children Down's syndrome patients 111.28 ± 76.02 SD pg/mL, children control 10.259 ± 1.403 SD pg/mL, p < 0.01; adult Down's syndrome patients 66.05 ± 50.38 SD pg/mL, adult control 6.57 ± 2.148 SD pg/mL, p > 0.05; old Down's syndrome patients 33.17 ± 31.77 SD pg/mL, old control 4.686 ± 1.756 SD pg/mL, p > 0.05)

Also MCP-1 serum level (fig 2C), displayed a statistically significative decrease in old Down's syndrome patients (103.78 ± 42.11 SD pg/mL) compared to children Down's syndrome patients (252.09 ± 91.06 SD pg/mL, p < 0.001) while it showed a not statistically significative decrease compared to adults Down's syndrome patients (174.99 ± 62.29 SD pg/mL, p > 0.05). DS patients levels differ form control subject of the same age (children Down's syndrome patients 252.09 ± 91.06 SD pg/mL, children control 125.20 ± 18.682 SD pg/mL, p < 0.001; adult Down's syndrome patients 174.99 ± 62.29 Sd pg/mL, adult control 89.167 ± 8.91 SD pg/mL, p > 0.05; old Down's syndrome patients 103.78 ± 42.11 SD pg/mL, old control 78.8 ± 9.911 SD pg/mL, p > 0.05)

Down's Syndrome patients maintain the same age related trend observed for the three molecules in old controls compared to adult and children ones. Furthermore, these ages related differences in serum levels of BDNF, IL-6 and MCP-1 are not statistically significative in control subjects, while in Down's syndrome patients are much more evident and statistically significative.

In particular, Fig 2A shows that level of BDNF displays a not statistically increase in old controls (11.245 ± 1.035 SD pg/mL) compared to adult controls (8.140 ± 1.274 SD pg/mL, (p > 0.05) and children controls (7.177 ± 0.469 SD pg/mL p > 0.05). Opposite trend is observed for IL-6 serum levels. Data shown in fig 2B indicate a not statistically decrease in old controls (4.686 ± 1.756 SD pg/mL) compared to adult controls (6.57 ± 2.148 SD pg/mL p > 0.05) and children controls (10.259 ± 1.403 SD pg/mL p > 0.05). Fig 2C displayed a not statistically decrease of MCP-1 levels in old controls (78.8 ± 9.311 SD pg/mL) compared to adult controls (89.167 ± 8.909 SD pg/mL p > 0.05) and children controls (125, 2 ± 18.682 SD pg/mL e p > 0.05).

The results are given as mean ± standard deviation (SD). Groups were compared by one-way analysis of variance. Linear regression analysis between experimental variables was also performed. Significance was taken as p < 0.05. Post-hoc comparisons within logical sets of means were performed using the Tukey-Kramer multiple comparison test (GraphPad Software Inc., San Diego CA).

## Discussion

DS is considered a condition with a low risk of clinical atherosclerosis and cardiovascular disease during adult and later life. An increment of molecules with functional roles in endothelial cell activation should raise the risk of atherosclerosis in DS. However, since DS is considered an atheroma-free model [2] and clinical investigations do not report any rise in the risk of cardiovascular disease in adult and elderly patients, DS patients constitute a still unsolved biological/clinical paradox [4]. Questions remain about the role of BDNF in atherosclerosis. We do not know why the level BDNF appears to be significantly depleted in humans with coronary atherosclerosis [10,11]. The fact that plasma BDNF levels are significantly reduced in the chronic, advanced [3] and acute stages [10] of coronary atherosclerosis points to a potential role of a deficit of this neurotrophin in atherosclerosis pathogenesis [12]. Neurotrophins are potentially important in atherosclerosis and related disorders [3,5]. A significant decrease in plasma NGF and BDNF was associated with the metabolic syndrome and atherosclerosis [13]. Age-related neurodegeneration associated with AD correlates with changes in BDNF expression too [8]. Recent investigations have looked into the hypothesis of a possible diurnal variation of BDNF circulating levels in human males. A correlation with cortisol circadian rhythm has also been sought, since both BDNF and cortisol are implicated in the maintenance of cerebral functions [14]. It has been suggested that BDNF might stimulate the production of proteins involved in cellular stress adaptation, growth and repair, neurogenesis, learning and memory and cell survival. There is also evidence that an increase of BDNF in either plasma or tissue has therapeutic potential [13]. Taking into account that BDNF is required for survival and function of hippocampal, cortical, basal forebrain, and entorhinal cortex neurons, and that in DS the neuropathological aspects are complex and include development of Alzheimer's disease (AD) in older age, we wanted to evaluate the change in BDNF levels in different age groups of Down's Syndrome patients, compared to the same age range healthy controls. Here we show an age-related increase of BDNF level DS patients serum, thus indicating a protective role of this molecule against atherosclerosis risk as observed by Nelson at al [15], showing that BDNF increase with age in healthy subject. Our data confirmed this age related increase in healthy control subjects, even if not statistically significative, and moreover it pointed out that this BDNF level increase is significatively higher in DS patients, in particular in old ones. In addition, among DS patients, this increase fits with an age related and progressive decrease of the proinflammatory and atherogenic cytokines IL-6 and MCP-1. Here we showed that plasma IL-6 and MCP-1 levels were elevated in DS compared to control subject, further supporting the notion of vessel dysfunction. This elevated level could not be ascribed to clinical inflammation, since DS patients had no clinically detectable pathological conditions. Therefore, it is likely that the plasma high level of pro-inflammatory cytokines in DS patients is due to endothelial activation without activation of immune responses. Elevated levels of IL-6 and intercellular adhesion molecules have been associated with endothelial dysfunction in various pathological conditions, such as atherosclerosis and its complications [9]. However, our results indicate that these elevated serum levels of the pro-inflammatory and atherogenic cytokines IL-6 and MCP-1 in DS patients, progressively decrease with age, in parallel with an age related increase of BDNF level.

## Conclusion

we suggest that in DS patients, elevated peripheral BDNF may protect against atherosclerosis, in particular in old DS people. In these patients, the higher incidence of dementia typically observed Down's syndrome old subjects, may induce a significant increase of plasma BDNF, as a protective response to prevent learning, memory functions and behavior degeneration.

## Abbreviations

BDNF: brain-derived neurotrophic factor; IL-6: interleukin-6; MCP-1: monocyte chemoattractant protein-1; DS: Down syndrome; AD: Alzheimer disease; NGF: nerve growth factor.

## Competing interests

The authors declare that they have no competing interests.

## Authors' contributions

GD carried out the protein array procedure and wrote the manuscript. EG participated to the experiment described in the text. FL participated to preparation of the materials used in the experiment and participated to the experiment described in the text. MMC senior author; conceived of the study, participated in its design and coordination. This work was supported by Banca Popolare di Milano Foundation Grant and by the Rector of University of Milan

Both authors read and approved the final manuscript.
